# ‘My Virtual Dream’: Collective Neurofeedback in an Immersive Art Environment

**DOI:** 10.1371/journal.pone.0130129

**Published:** 2015-07-08

**Authors:** Natasha Kovacevic, Petra Ritter, William Tays, Sylvain Moreno, Anthony Randal McIntosh

**Affiliations:** 1 Rotman Research Institute, Baycrest Centre, Toronto, Ontario, Canada; 2 Max Planck Institute for Cognitive and Brain Science, Leipzig, Germany; 3 Department of Neurology, Charité –Universitätsmedizin Berlin, Berlin, Germany; 4 Department of Psychology, University of Toronto, Ontario, Canada; Interdisciplinary Center (IDC) Herzliya, ISRAEL

## Abstract

While human brains are specialized for complex and variable real world tasks, most neuroscience studies reduce environmental complexity, which limits the range of behaviours that can be explored. Motivated to overcome this limitation, we conducted a large-scale experiment with electroencephalography (EEG) based brain-computer interface (BCI) technology as part of an immersive multi-media science-art installation. Data from 523 participants were collected in a single night. The exploratory experiment was designed as a collective computer game where players manipulated mental states of relaxation and concentration with neurofeedback targeting modulation of relative spectral power in alpha and beta frequency ranges. Besides validating robust time-of-night effects, gender differences and distinct spectral power patterns for the two mental states, our results also show differences in neurofeedback learning outcome. The unusually large sample size allowed us to detect unprecedented speed of learning changes in the power spectrum (~ 1 min). Moreover, we found that participants' baseline brain activity predicted subsequent neurofeedback beta training, indicating state-dependent learning. Besides revealing these training effects, which are relevant for BCI applications, our results validate a novel platform engaging art and science and fostering the understanding of brains under natural conditions.

## Introduction

Advances in cognitive neuroscience are increasingly engaging wider audiences. While traditional experiments in controlled laboratory conditions and simple paradigms have contributed significant insights into brain function, it is also widely recognized that our understanding of complex cognitive phenomena requires similarly complex realistic environments [1–4]. We present here an electroencephalography (EEG) based brain-computer-interface (BCI) experiment that was conducted as a part of a public art installation, ‘My Virtual Dream’, during Toronto’s Nuit Blanche art festival on October 5, 2013 (scotiabanknuitblanche.ca). Our data acquisition framework was innovative at multiple levels: a) festival visitors were intrinsically motivated to interact collectively with the large-scale immersive virtual environment; b) EEG-based BCI data were collected from large number participants (N = 523) in a single night; c) the simultaneous multi-subject (N = 20) EEG-BCI study occurred in a gaming environment for individual and collective neurofeedback targeting two brain states, relaxation and concentration.

Besides validating the novel experimental setup, our focus was to explore participants’ ability to rapidly learn to control their brain states in a complex environment. This, together with art-exhibition related criteria, guided the experimental design. The controlled neurofeedback participants received lasted less than 7 min, which is much shorter than typical neurofeedback training experiments [5]. This was motivated by our hypothesis that the neurofeedback effects could be detected early in training and that the large sample size would provide sufficient statistical power to reveal them.

The technological maturity and aesthetic sophistication of multi-media, gaming, and virtual reality position these platforms as perfect partners for neuroscience. Also EEG is expanding into uses outside the laboratory ([6–10]) through therapeutic neurofeedback interventions and BCI technology (e.g. [11–15]) as well as in consumer products such as wearable devices for neurogaming, self-monitoring and self-optimization. Various BCI-based neurofeedback protocols present promising practical utility with respect to learning, attention, and even creativity [16].

The utility of BCI applications learning is enhanced if one can learn how to modulate one’s brain activity in as little time as possible. Learning is associated with functional and structural changes in the brain [17–22]. Despite continuous reorganization on the synaptic scale, large-scale effects require time to manifest. Structural changes can be detected with non-invasive imaging after 45 min to few hours of training [19], [23]. Sensory stimulation protocols yield persisting reorganization of coupling between distributed brain areas after 30 min of stimulation [16]. In terms of cognitive performance, singular neurofeedback training sessions can mediate significant changes [24], [25]. In the setup of My Virtual Dream we aimed to provide an inspirational learning environment and at the same time to achieve enough statistical power to detect subtle early, learning-related changes of brain activity due to collective neurofeedback training.

**Fig 1:**
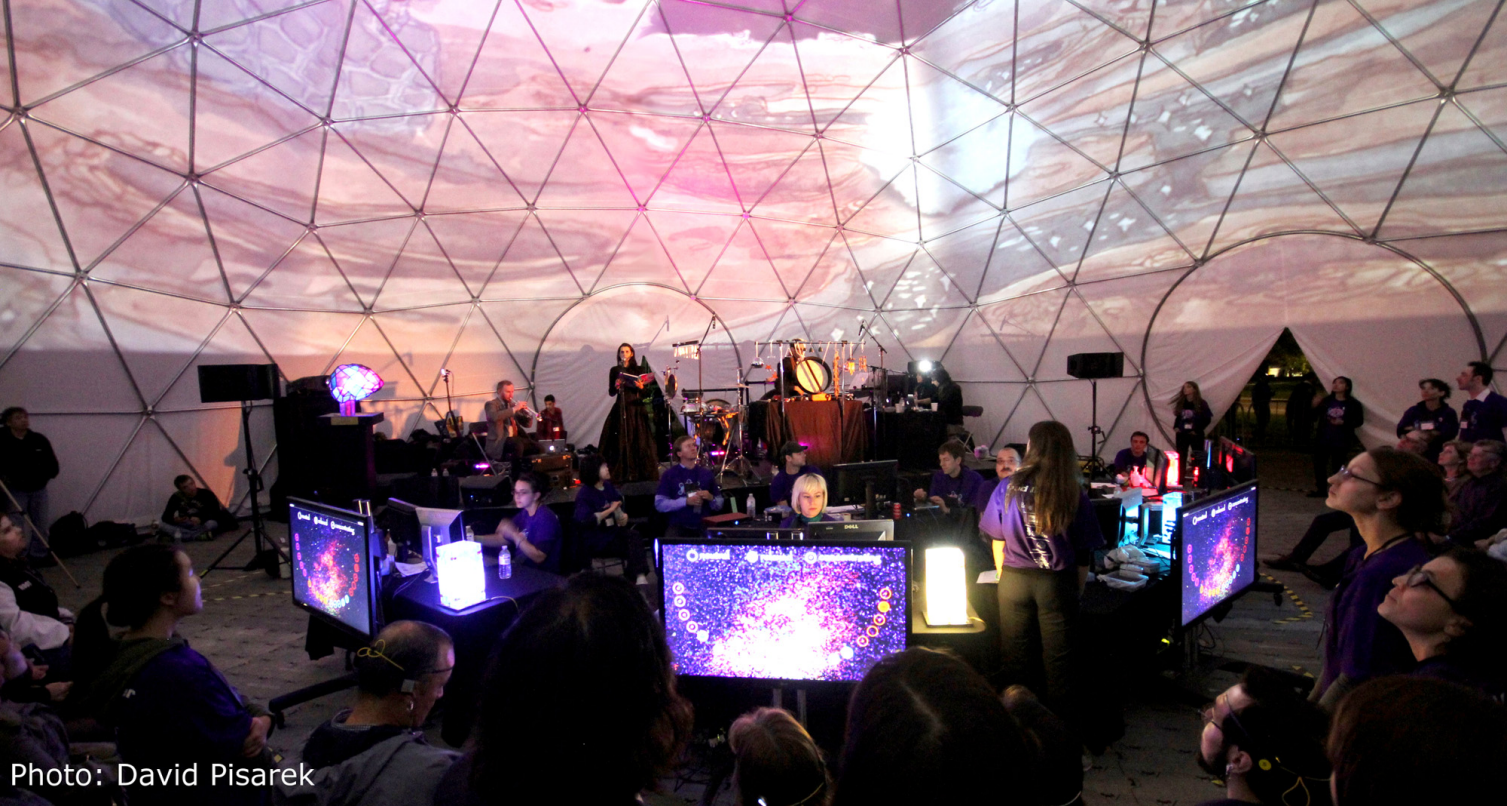
My Virtual Dream: the ‘dreamery’ and the stage. In front of an audience, twenty participants at a time experienced a two-part interaction within the dome. Based on the collective neurofeedback of all 20 participants, the ‘dreamers’, artistic video animations were projected on the 360° surface of the semi-transparent dome and soundscapes were generated based on a pre-recorded sound library and improvisations from live musicians.

The inspiration for the installation came from The Virtual Brain project (http://thevirtualbrain.org/tvb/zwei), a neuroinformatics platform for full brain computer simulations [26–28]. My Virtual Dream, envisioned as a ‘portal into the mind’, was an immersive audiovisual environment: an 18 m geodesic dome illuminated by dream-like artistic visuals and soundscapes driven by collective brain waves (Fig 1). Twenty participants at a time experienced a two-part interaction in the dome, in front of 50 spectators. In the first part the participants played a collective game, which required them to alternate between states of relaxation and concentration as defined by their own EEG recording. The game lasted 6.5 min. After the game, the participants proceeded into the ‘dream experience’. There were four thematic libraries of ‘dream snippets’: animated video clips paired with pre-recorded musical clips. Each ‘dream’ accessed one of the four libraries and composed the audio-video assets in real time based on the collective neurofeedback of all 20 dreamers. The dreams were projected onto the entire 360° surface of the dome. The dream experience was further enhanced with live improvisations from three musicians inspired by the brain-triggered dream sequences. In My Virtual Dream, participants simultaneously reacted to, and changed the external environment. An overview and the of the dreamer’s experience are captured in S1 Movie. For this article we considered only the data from the game sequence.

## Materials and Methods

### Subjects

The event started at 7 pm on October 5, 2013 and lasted 12 hours. Throughout the night we conducted 29 sessions, yielding a total of 577 participants. Our volunteers were instructed to reject anyone who was visibly intoxicated. Average wait time in the lineup was 2 hours. We collected age and sex of participants as demographic data. The minimum age for participation was 18 years. In some cases EEG data was not correctly recorded (too short or failed neurofeedback calibration) and for two sessions demographic data was not collected by omission. From the entire sample of 577 data sets, we extracted 523 fully usable data sets (314 females, 209 males). Ages ranged from 18 to 89 years, with an average age of 31.1(SD: 13.9).

### Ethics statement

The experiment was approved by the Research Ethics Board at Baycrest, and all participants gave informed written consent for their participation (following the guidelines of the Research Ethics Board at Baycrest and the University of Toronto).

With respect to the filming of the event, the following Disclaimer was posted at all public entrances to the event area, as approved by the Research Ethics Board at Baycrest:

*Please be advised that by attending this event*, *you enter an area where photography*, *audio and video recording will occur*. *Portions of today's event will be posted on promotional and educational material (digital and print) created by Baycrest and University of Toronto*. *By entering the event premises*, *you consent to*: *photography*, *audio recording*, *video recording and its/their release*, *publication*, *or reproduction to be used for inclusion on websites and print publications by Baycrest and the University of Toronto and its affiliates and representatives*. *You release Baycrest and the University of Toronto*, *its officers and employees*, *and each and all persons involved*, *from any liability connected with the taking*, *recording*, *digitizing*, *or publication of photographs*, *computer images*, *video and/or or sound recordings*. *By entering the event premises*, *you waive all rights to any claims for payment or royalties in connection with any streaming*, *or other publication of these materials*, *or other publication regardless of whether a fee for admission is charged*. *You also waive any right to inspect or approve any photo*, *video*, *or audio recording taken in these premises or the person or entity designated to do so*. *You have been fully informed of your consent*, *waiver of liability*, *and release before entering the event*. *If you do not want us to use a photo or video of you*, *please let us know when you arrive at the event or e-mail* *myvirtualdream@research.baycrest.org*

### EEG acquisition

EEG data were collected using 20 wireless ‘Muse’ headsets which were provided by Interaxon (http://interaxon.ca/). The headsets were manually assembled prototypes and specially fitted with long lasting batteries to accommodate the requirements for our 12 hr event. Each headset was first paired to a computer and assigned a Bluetooth serial port. Next, we initiated an instance of Muse Connector, an accompanying interface software package, which collects data from the Bluetooth serial port and forwards packets of EEG data in Open Sound Control (OSC) format via User Datagram Protocol (UDP). The headsets were equipped with seven sensors placed at mastoids (ear clips) and frontal regions Fp1 and Fp2. The remaining three sensors were used as electrical reference. Muse headsets initially oversample EEG and subsequently downsamples it to yield a selectable output sampling rate from 220 Hz to 500 Hz, with 2uV (RMS) noise. The input range of AC coupled signal (low cut-off at 1 Hz) is 2 mV.

### Procedure

Sixty volunteers assisted with instructions, setup and supervision of the experiment. Before entering the dome, participants received a brief 10 min overview of the EEG headset technology and the game they were about to play. The participants were made aware that the data would be anonymized and collected for scientific purposes. They were given the option to disallow use of their EEG recordings. This option was chosen in only four cases.

A session started with the 20 participants being seated in a semicircle in front of the stage and divided into four groups (‘pods’) of five. For each pod, EEG data were collected simultaneously from all 5 wireless headsets and streamed into a collective neurofeedback game displayed on a 48’ LCD screen. The display was mirrored on a computer screen facing away from participants and towards the center of the semicircle where the main network switch and the central hub computer were located and My Virtual Dream staff was seated for supervision. The master of ceremonies coached the participants through the progression of the game.

### Neurofeedback and game experience

Neurofeedback was implemented with custom-built software and was based on relative spectral power (*RSP*) in alpha (8–12 Hz) and beta (18–30 Hz) bands. The tutorial part of the game was used for personal calibration, i.e., to determine the upper and lower thresholds for both bands of interest for each individual. Based on the individual thresholds, four types of neurofeedback messages were sent to the game: whenever relative alpha power was higher (lower) than the upper(lower) alpha threshold, the driver sent *a+* (*a-*) message, and analogously *b+* (*b-*) for relative beta power (see details below).

**Fig 2:**
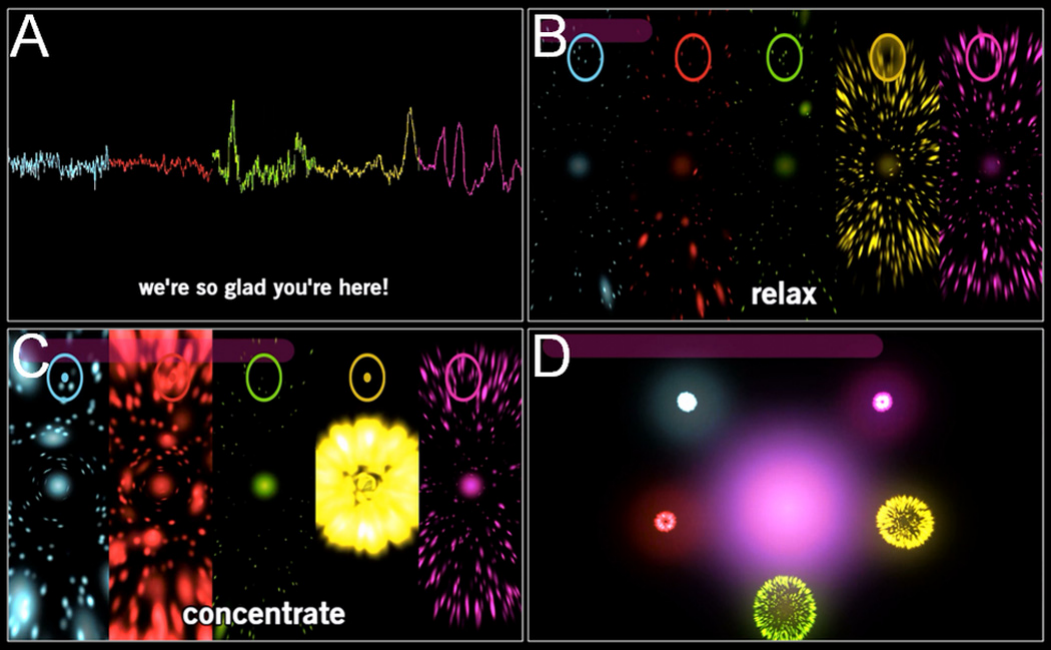
Game screenshots. **(A)** EEG data observation and welcome messages. Players are ordered from left to right. **(B)** Solo relax. Number of particles indicates cumulative relaxation result (e.g., players 4 and 5 accumulated large particle cloud). Ovals on the top of the screen represent additional feedback such that light shading of the oval signifies *a+* state (e.g. player 4). **(C)** Solo concentrate. Brightness of particles indicates cumulative concentration result (e.g., players 1, 2 and 4 have good result). Dot inside an oval indicates that player is in *b+* state (e.g., players 1 and 4). **(D)** Group game, guided and freestyle.

**Fig 3:**
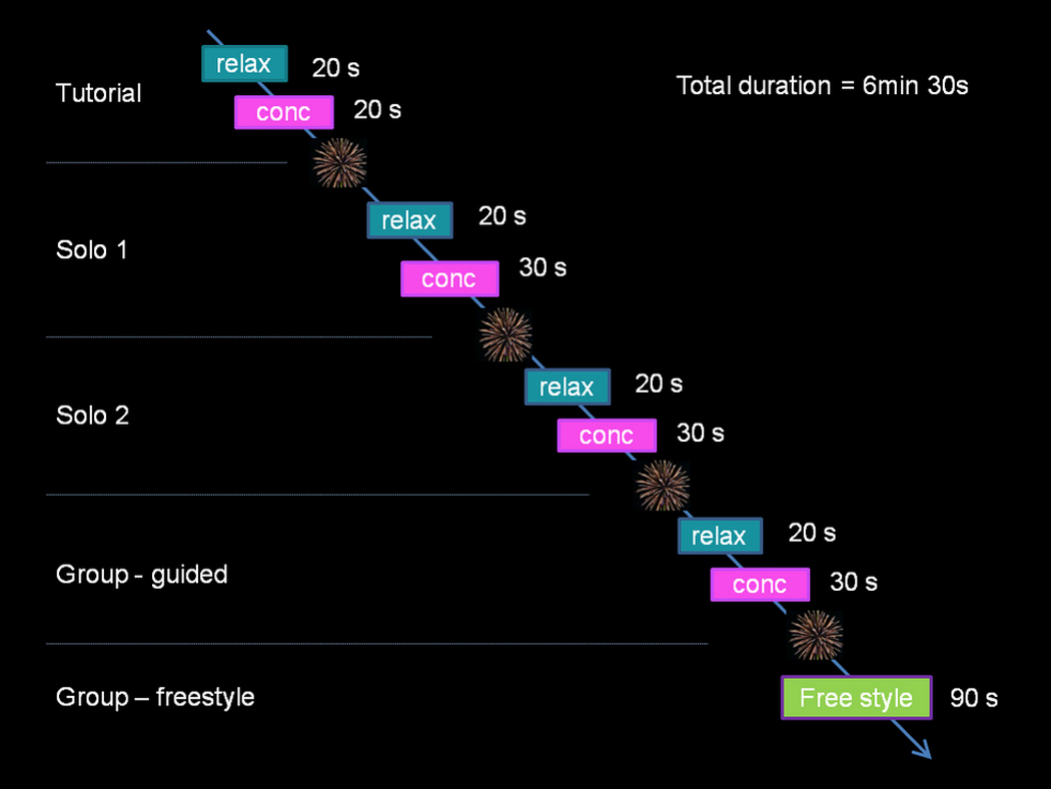
Game timeline. Each phase of the game ended with fireworks display, size and brightness of which were determined by the performance of the participants. Total duration of the game was 6.5 min. In the Tutorial individual thresholds for alpha and beta were estimated based on guided ‘relax’ and ‘concentrate’ conditions. Participants obtained individual visual feedback on their performance to either increase alpha or beta. Solo 1 and 2 games were qualitatively identical with the tutorial, however individual thresholds were used. During the ‘group-guided’ game each ‘pod’ obtained feedback about the collective performance in addition to the individual feedback. In the ‘Group—freestyle’ period, participants did not obtain specific instructions other than to attempt to synchronize as a group by targeting the same brain state.

Screenshots from different phases of the game are shown in Fig 2 and full game video capture is provided in S2 Movie. The game experience was divided into 6 phases:

1. *EEG data observation*. The screen was split into five color-coded vertical strips, each displaying ongoing EEG data of the participants. This served two purposes: to enable assistants to adjust the headsets if necessary and for participants to identify their own brain waves (Fig 2A). During this phase, participants were encouraged to blink and move their face muscles and observe the effects on the ongoing signal. The intention was to convince the participants that the displays were truly driven by their brain signals in real time. Assistants who were operating the software on pod computers waited until they received a hand signal from the central hub to indicate that everyone’s data had stabilized and to start the game. Thus all 4 pods would start the game at approximately the same time. From here onwards, the game unfolded automatically according to the timeline shown in Fig 3.
2. *Welcome messages*. At the very beginning of the game, before the onset of the tutorial, participants saw several welcome messages for a total duration of 3.5 s. This interval was used as the baseline for predicting subsequent beta learning in the post-hoc analysis (see Results).
3. *Tutorial*. The screen was still divided into five vertical strips and an instruction message ‘relax’ appeared and remained for 20 s. During this time the visual feedback was based on alpha messages. The participants ‘gathered particles’ into an orb, and were rewarded with more particles when exhibiting sustained increased alpha power (Fig 2B). The behind-the-scenes alpha counter increased/decreased with the influx of *a+*/*a-* messages, and was clamped to values > = 0. Player received positive feedback via more particles whenever the counter surpassed a positive threshold, after which point the counter was reset to 0. There was no negative feedback, i.e., the number of particles never decreased. Subsequently, the instruction ‘concentrate’ appeared on the screen and remained for 20 s. During this time participants ‘condensed energy’, that is, previously gathered particles brightened and intensified according to the influx of *b+/b-* messages (Fig 2C). In this case, the behind-the-scenes beta counter operated in a similar fashion by rewarding sustained high beta power. After the ‘concentrate’ period, each participant’s ‘energy orb’ was launched into a firework. The height of the firework reflected the high alpha achievement during the ‘relax’ condition, whereas the brightness of the firework indicated the participant’s performance with high beta states during the ‘concentrate’ condition. No scores were displayed at any time as we aimed to create a pleasant and collective experience and downplay the potential for competitiveness. During the tutorial, alpha and beta messages were generated using default thresholds. However, the data collected during the tutorial were stored and subsequently analyzed. Individual thresholds were calculated from the mean alpha and beta relative power during the tutorial: lower and upper alpha (beta) thresholds were set as 0.9 (1.0) and 1.1 (1.2) of the mean alpha (beta) power. The remainder of the game was driven using the customized thresholds.
4. *Solo 1 and 2 games*. These two stages of the game were qualitatively identical with the tutorial, however individual neurofeedback thresholds were used and the ‘concentrate’ conditions lasted 30 s.
5. *Group—guided game*. Here each ‘pod’ obtained feedback about the collective performance in addition to the individual feedback. This was realized by moving the particle orbs of individual participants into a semi-circular shape surrounding a ‘group orb’ at the center of the screen (Fig 2D). The logic was the same as in solo fireworks, with 20 s of relaxation followed by 30 s of concentration. In this case however, the group’s orb increased in size whenever 3 or more participants were in the target state. The idea here was to introduce the participants to the common goal and the corresponding visual feedback. After the ‘concentrate’ condition, all orbs merged together and launched group fireworks, the height and brightness of which was reflective of the pod’s performance.
6. *Group—freestyle game*. This part of the game was similar to the guided group game, except that there were no specific instructions and it lasted 90 s. Before the start, the participants were instructed to synchronize with other members of the pod, that is, to either concentrate or relax, whichever appeared appropriate at any given time. The group orb in the middle increased in size whenever three or more participants were in the same state. The total number of synchrony events determined the size and brightness of the final group fireworks.

### Data preprocessing and *RSP*

#### Bluetooth timing rectification

The BCI software recorded the incoming EEG data with timestamps, while the game recorded timestamps for condition onsets and offsets. In theory, this would allow us to epoch the data, i.e. extract condition-specific portions of EEG time series. However, we detected timing and sampling issues with Bluetooth headsets. For each EEG data set, we estimated the headset’s EEG sampling rate by dividing the number of recorded data points with the time difference between the first and the last time stamp. Though the target output sampling rate was chosen to be 220 Hz, all headsets had higher estimated sampling rates with a mean of 229.5 Hz (SD: -11.8 Hz). The estimated individual headset sampling rate was stable across recordings, with an SD of 0.34 Hz on average. The estimates were based on 8 min of recording or more. However, the data came in bursts and at a slightly variable sampling rate, due to the limitations of Bluetooth technology. Bursts of data with the same time stamps consisted of 33 data points, on average. We assumed that accurate timestamps corresponded to endpoints of the bursts, so for the data acquired between two consecutive bursts, we interpolated intermediate timestamps as if acquired with even sampling. Because our conditions were at least 20 s long, this was acceptable accuracy in determining the condition onsets and offsets.

#### Epoching

We defined 9 conditions in accordance with the game timeline. The condition names and durations were: Tutorial-relax (20 s), Tutorial-concentrates (20 s), Solo1-relax (20 s), Solo1-concentrate (30 s), Solo2-relax (20 s), Solo2-concentrate (30 s), Group-guided-relax (20 s), Group-guided-concentrate (30 s), and Group-freestyle (90 s). The freestyle condition was not analyzed since it was distinctly longer and different than all other conditions. Also, because some conditions were 30 s long, we always utilized only the first 20 s of each condition segment. Condition specific segments were resampled to 256 Hz. For subsequent data analysis we split the long segments into a series of overlapping 1s epochs, with 100 ms overlaps. For 20 s of continuous data resampled to 256 Hz this procedure resulted in 211 epochs. Temporal mean was subtracted from each epoch.

#### Re-referencing and artifact rejection

**Fig 4:**
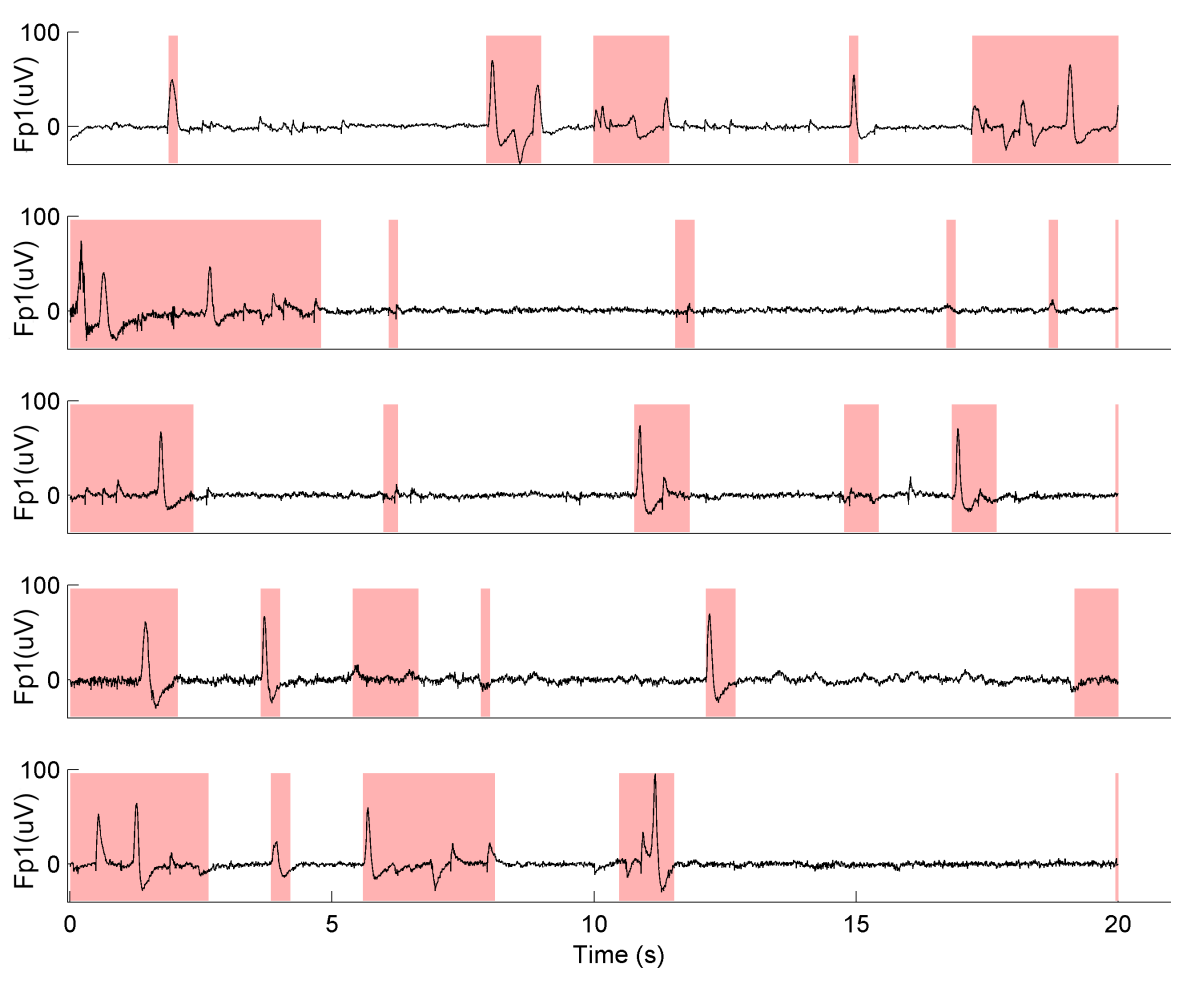
Results of the automated artifact rejection procedure. EEG traces from the left frontal channel of 5 randomly selected participants are shown during the first 20 s of the Solo 1-concentrate condition. Shaded areas indicate rejected intervals.

The four channel data were re-referenced to mastoids by subtracting the average signal from ear electrodes. This resulted in two frontal channels and one average ear channel. Given the size of the data set (N = 523) we opted for an automatic procedure for artifact rejection. The procedure sorted epochs within each condition according to the sum of square amplitudes, and for each subject the first 100 epochs with the lowest total amplitude were kept for further analysis. Visual inspection of randomly selected epochs from the entire data collection confirmed that automatically selected EEG data were reasonably clean (Fig 4). Data from two frontal channels were used in all subsequent analyses.

#### RSP

To obtain *RSP* estimate for each condition we calculated relative spectral power in the 1–50 Hz frequency range. The power spectrum *P* was calculated for each frontal channel. Next, relative power for each frequency *i* = 1:50 Hz was calculated:$P_{rel}\left(i\right)=P(i)/\sum_{j=3}^{50}P(j)\text{.}$ Final *RSP* estimate was obtained by averaging *P*_*rel*_ across epochs and taking *log* transform with natural logarithm.

#### Baseline *RSP*

To estimate baseline RSP we used the EEG data prior to any training from a 3.5 s interval during the welcome messages at the very start of the game. Preprocessing was similar as for the relaxation and concentration conditions, only here we compensated for shorter interval by epoching with only 20 ms overlap. This produced 406 epochs, and we obtained RSP estimate by averaging over 100 epochs with the lowest total amplitude, then taking the natural logarithm.

#### Multivariate statistics

Group and condition effects were analyzed by Partial Least Squares (PLS), a multivariate statistical toolbox [29], [30]. Here we describe briefly two types of PLS: task PLS and behavioral PLS. Task PLS computes a covariance matrix of brain data (e.g., RSP values across channels and frequencies) and a set of design contrasts (e.g., group and/or condition effects). In the data driven implementation, the design contrasts are obtained by performing singular value decomposition (SVD) on the covariance matrix into a set of orthogonal latent variables, LV’s. Each LV consists of a pair of optimally related patterns: a left singular vector (brain pattern) and a right singular vector (group/condition effect), and a singular value (size of the effect). In the hypothesis driven implementation, the user selects a set of design contrasts, which are then projected onto the brain data. In both implementations, the reliability of the relationship between the paired brain patterns and design contrasts is assessed using split-half resampling [30], which produces two p-values: pbrain estimating the reliability of the brain pattern given the design contrast, and pdesign estimating the reliability of the design contrast given the brain pattern. In the present study, effects are considered reliable if maximum of the two p values, denoted maxp, is < .05. Additionally, PLS assesses reliability of individual components of the brain patterns via bootstrapping resampling of participants with replacement. For each brain data component (e.g., channel at a specific frequency) PLS calculates a bootstrap ratio as a component’s loading divided by standard error of the loading across bootstrap samples, akin to a z-score. Bootstrap ratio was considered stable when its absolute value was greater than Sidak’s threshold [31]. Behavioral PLS computes a correlation between the brain data matrix and some dependent measure(s) such as behavior or age. In this case SVD decomposes the correlation matrix, while the reliability of the resulting latent variables (brain patterns with associated correlation patterns) is examined in the same way as in task PLS. In all PLS analyses we used 100 permutations, 100 split-half samples and 500 bootstrap samples. For each LV participant scores are obtained by multiplying the brain data for each condition (in our case RSP values) by the left singular vector. Group/condition effect is depicted by mean participant scores, together with confidence intervals (CI’s) based on bootstrap resampling.

#### Global effects

In the confirmatory analyses we were interested in global patterns of correlation between the brain data and demographic variables, regardless of conditions. We thus folded all condition specific RSP measurements as observations. In other words, for each participant, the brain data included the full collection of RSP values across all conditions together. The correlation structure between the brain data and each of the demographic variables (age, sex and time-of-night) was analyzed using behavioral PLS.

#### Headset effect

Since wireless headsets were manually assembled prototypes, we first investigated differences between headsets. We divided subjects into 20 groups, according to the headset, and ran task PLS for group differences. The analysis detected several strong and reliable effects (results not shown). We considered the headset effects as nuisance variables and regressed them from all subsequent analyses. This was done by extracting participant scores for all non-zero latent variables and regressing them out from *RSP* data across subjects.

#### Time-of-night effect

Since data acquisition started at 7pm and ended at 7am on the following morning, we expected to see an effect of sleep deprivation. Although we did not collect any sleep related data from the participants, it is reasonable to assume that sleep deprivation increased as night progressed. To probe this effect we correlated EEG data with session number 1–29, where 1 corresponded to the first session at 7pm and 29 corresponded to the last session around 6:35 am. We then analyzed the correlation between *RSP* and time-of-night using behavioral PLS. Once again, we considered this effect as nuisance variable and regressed out the session number from brain data in subsequent analyses.

#### Age effect

We used the entire sample to correlate *RSP* and age and applied behavioral PLS.

#### Sex effect

Sex differences in *RSP* were analyzed as a group effect with task PLS. Prior to the analysis, we regressed out headset and time-of-night effects. We also regressed age and repeated the analysis a second time. Both analyses showed virtually identical results.

### Neurofeedback effects

#### Relaxation vs. concentration

To examine the main effect of relaxation vs. concentration, we ran data driven task PLS on *RSP* values across all guided conditions: Tutorial-relax, Tutorial-concentrate, Solo1-relax, Solo1-concentrate, Solo2-relax, Solo2-concentrate, Group-guided-relax, Group-guided-concentrate.

#### Training effects

For the analysis of training effects for concentration and relaxation, the data were first cleared of nuisance variables related to headset, time-of-night, and age, as described above. We ran two data driven task PLS analyses, first with Tutorial-relax, Solo1-relax, and Solo2-relax conditions, and second with Tutorial-concentrate, Solo1-concentrate, and Solo2-concnetrate conditions.

To examine the impact of sample size on detectability of training effects, we repeated the analyses with lower number of randomly selected participants. We focused on the concentration training effect and performed a series of PLS analyses with the number of participants, *N*, ranging from 10 to 500 in multiples of 10. For each *N*, participants were chosen at random, and we tested a fixed contrast coding for the training effect, [-1 0 1], corresponding to Tutorial-concentrate, Solo1-concentrate, and Solo2-concentrate conditions. Randomization was repeated 50 times and mean *maxp* was calculated as an estimate of the training effect reliability for *N* participants.

#### Neurofeedback performance measures

We defined two performance measures based on the ability of the participants to maintain the desired state. Relaxation maintenance, i.e., *alpha performance aP*, was operationally defined as the ratio of the total number of *a+* states divided by the total number of *a-* states during a relax condition, where *a+* (*a-*) correspond to high (low) power in alpha range. This provided an estimate of the player’s maintenance of high alpha power. Concentration maintenance, i.e., *beta performance bP*, was defined analogously and good beta performance during concentration blocks was associated with high values of *bP*. Both measures were evaluated across all conditions.

Since we found large variations in beta learning (see Results), we divided subjects into two groups, *beta learners* and *beta non-learners* and used task PLS to test for group differences in baseline *RSP*. We also tested the differences in *aP* and *bP* between beta learners and non-learners by running two separate PLS analyses across all conditions.

## Results

### Neurofeedback learning of the target brain states

**Fig 5:**
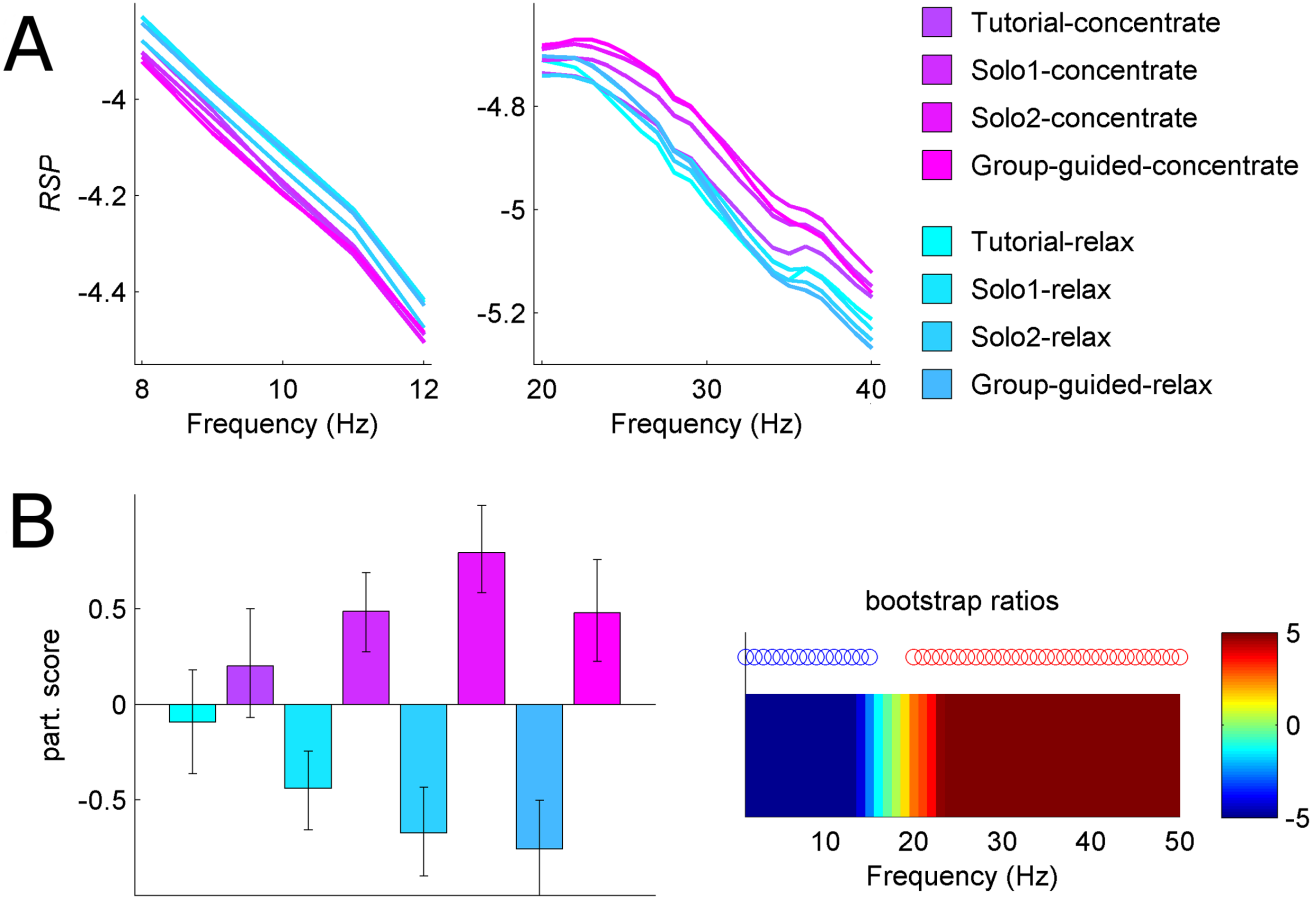
Relaxation vs. concentration states (example left frontal channel). **(A)** Mean *RSP* curves across 8 guided conditions in alpha and beta frequency ranges. **(B)** Left: mean participant scores with respect to the main effect of relaxation vs. concentration across 1–50 Hz frequency range. Error bars indicate 95% confidence intervals (CI) Right: frequency pattern associated with the main effect. Frequencies with reliable positive (negative) bootstrap ratios exhibited greater (lower) *RSP* for concentration conditions compared to relaxation conditions and are indicated by red (blue) circles. As hypothesized, concentrate conditions showed more power in beta range and less power in alpha range, compared to relax conditions.

The omnibus data driven analysis of condition effects in relative spectral power identified the dominant reliable effect distinguishing relaxation vs. concentration blocks (Fig 5). As expected [32], power in beta range was higher for concentrate conditions, whereas the power in the alpha range was higher in relax conditions. Moreover, in the solo games the differentiation between relaxation and concentration increased with training and reached maximum difference during Solo2.

**Fig 6:**
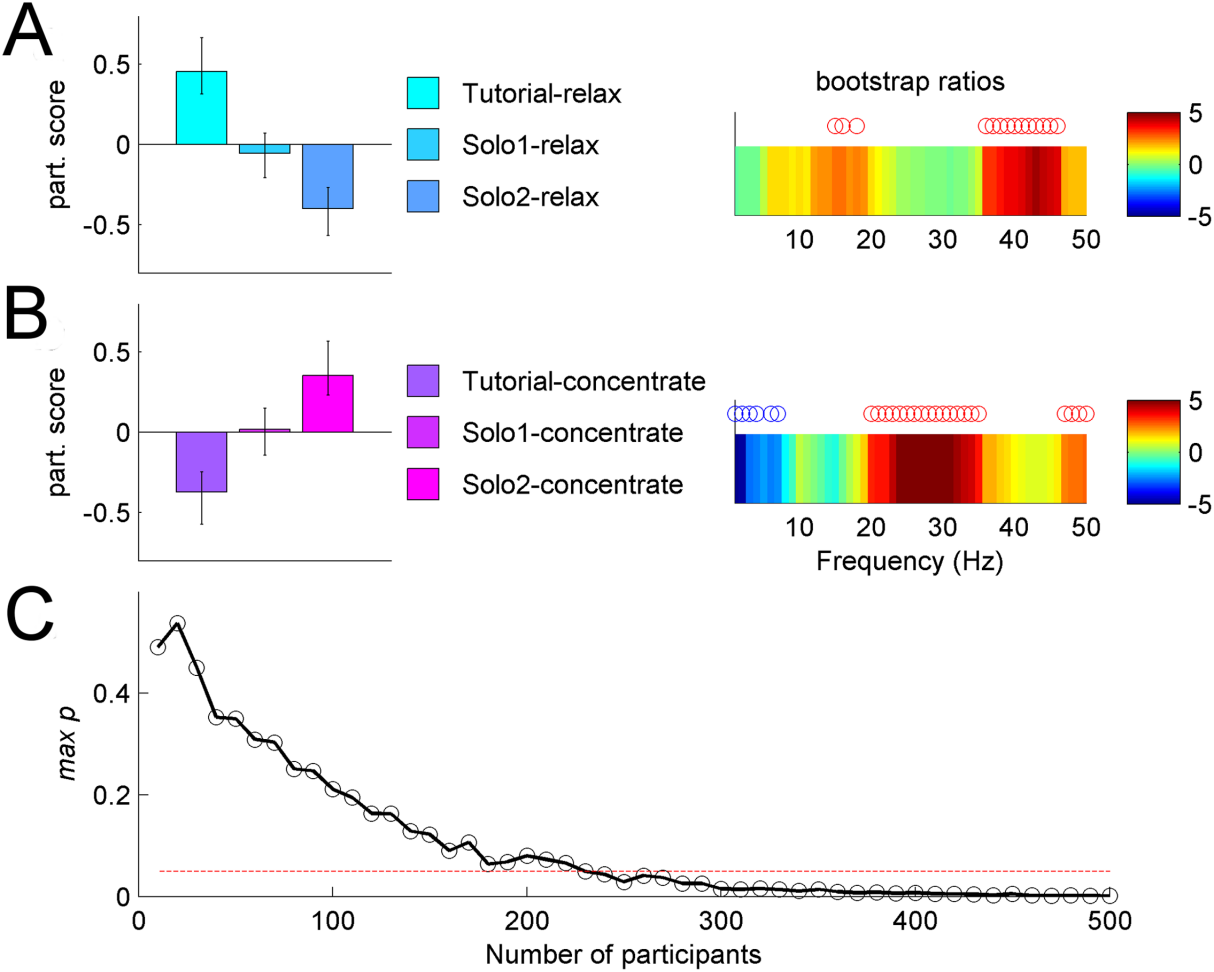
Training effects on brain states (example left frontal channel). **(A)** Relaxation training effect. Left: mean participant scores with respect to the relaxation training effect, with error bars representing 95% CI. Right: associated frequency pattern with reliable positive (negative) bootstrap ratios indicated by red (blue) circles. Overall effect of relaxation training is a decrease in 17–18 Hz and 35–45 Hz frequency range. **(B)** Concentration training effect. Left: mean participant scores with error bars representing 95% CI. Right: associated frequency pattern with reliable positive (negative) bootstrap ratios indicated by red (blue) circles. Overall effect of concentration training is an increase in beta power (20–40 Hz frequency range) and a decrease in delta power (<3 Hz). **(C)** Reliability of concentration training effect across a series of analyses with varying number of participants, N. For each N we plotted mean estimate of the reliability measure *maxp*. Reliable results (*maxp* < 0.05, i.e., below the red dotted line) are consistently obtained when the number of participants is >>200.

We further investigated training effects for each target brain state separately. The analysis of the three solo relaxation conditions (Fig 6A) showed gradual decreases in mid range and high frequencies (8–20 Hz and 35–45 Hz). By contrast, concentration training (Fig 5B) revealed gradual increases in beta range and decreases in low frequencies (< 3 Hz). In other words, participants were learning to modulate their relative spectral power within only 60 s and 80 s training periods, for relaxation and concentration respectively (the total time for the three solo conditions).

Pilot experiments leading to the public event consistently identified the main relaxation vs. concentration effect, even with very small numbers of participants (N< = 10, data not shown). Based on this, we hypothesized that early yet subtle brain activity changes associated with short neurofeedback training protocol would become detectable with larger sample sizes if present. We investigated the impact of the sample size on the statistical reliability for the concentration training effect. Results shown in Fig 6C confirm that training effects are reliable exclusively for large sample sizes N >>200.

### Persistent neurofeedback learning effects

**Fig 7:**
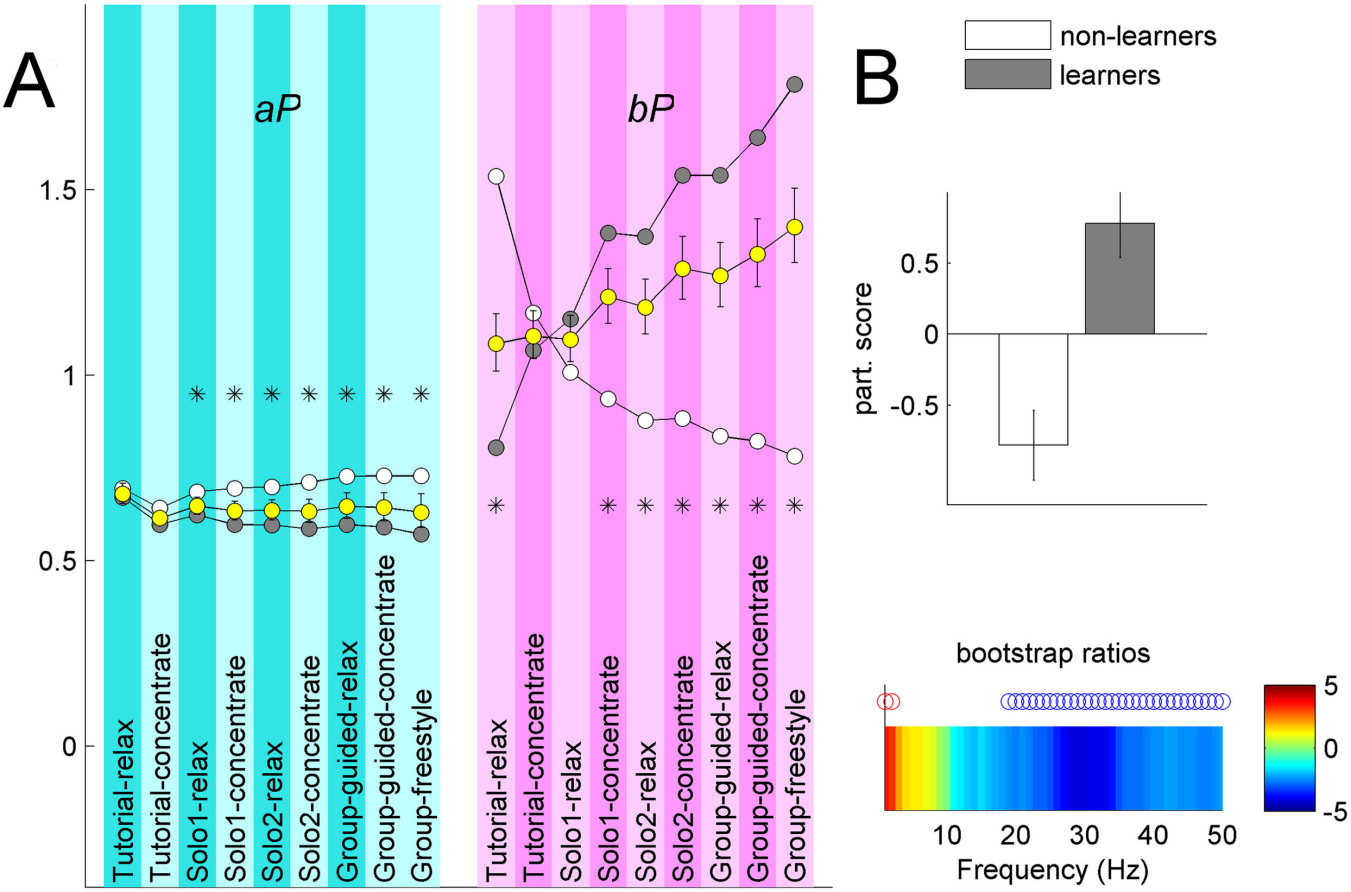
Neurofeedback performance measures. **(A)** Group mean alpha performance *aP* and beta performance *bP* for all participants taken together (yellow bullets), with 95% CI’s shown as error bars, beta learners (gray bullets) and non-learners (white bullets). Conditions where neurofeedback did not depend on the respective band of interest are shown in desaturated color. Black asterisks indicate conditions which expressed reliable PLS difference between learners and non-learners. **(B)** Analysis of differences in baseline *RSP* between beta learners and non-learners. Top: mean participant scores with error bars representing 95% CI. Bottom: associated frequency pattern for the left frontal channel with reliable positive (negative) bootstrap ratios indicated by red (blue) circles. High power in delta range and low power in beta/gamma range during baseline predicted subsequent beta learning.

We evaluated the two performance measures across all conditions. Besides the overall difference between *aP* and *bP* due to different threshold settings, we observed striking training-related differences between the two performance measures: while *aP* mostly stayed at the baseline level throughout the game, *bP* steadily increased, though with large standard error actross the entire sample (Fig 7A). Percent increase in *bP* from the start to the end, i.e., from Tutorial-relax to Group-freestyle was taken as the beta training effect, *bTE*. The mean value of *bTE* was 87% (SE: 9%). To better understand the large variability in *bTE* we split the sample into two groups, ‘beta learners’ and ‘beta non-learners’, which we defined according to *bTE* > 0 and *bTE* < = 0. There were 201 non-learners and 322 learners. The mean *bTE* for learners and non-learners were 169% (SE: 13%) and -44% (SE: 2%), respectively, and were statstically different according to the t-test at p<0.001.

For learners, effects of concentration training in beta band persisted even during the non-concentration blocks, suggesting learning-related changes in the brain. By contrast, beta non-learners showed steady *bP* decrease. PLS analyses of group differences in performance measures, *aP* and *bP*, produced reliable latent variables (Fig 7A). The effect size for *bP*, however, was 5.8 times greater than for *aP*.

Given the striking difference in the learning trajectories for beta learners and non-learners we investigated whether power spectrum at baseline (time interval directly preceding the onset of the neurofeedback training) could predict beta learning. Indeed, the two groups were found to be statistically different at baseline such that learners had less relative power in beta/gamma range and more power in delta range, compared to non-learners (Fig 7B). No relationship between beta learning and demographic factors were found.

### Time-of-night and demographic factors

**Fig 8:**
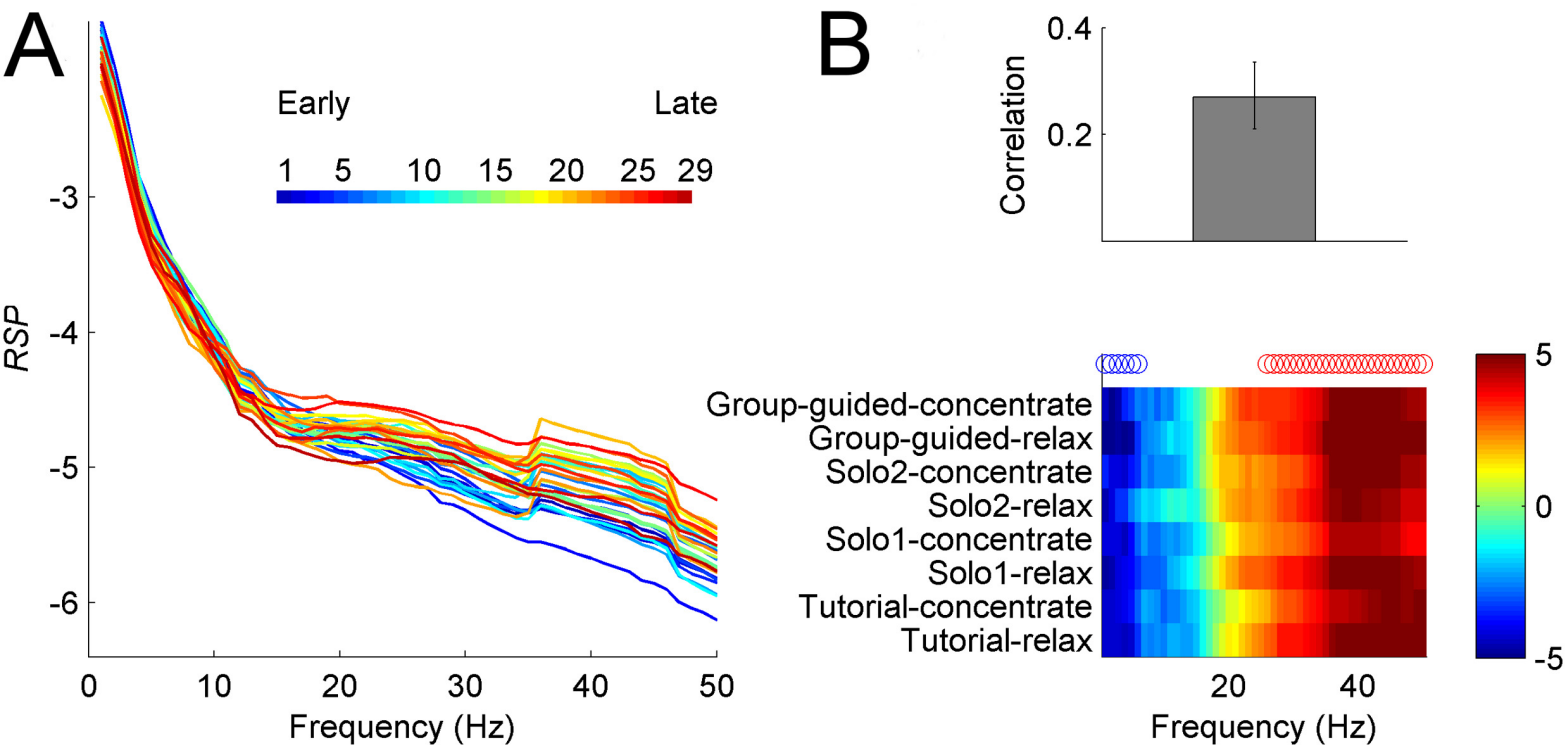
Time-of-night effect (example left frontal channel). **(A)** Mean *RSP* curves from 29 sessions during Tutorial-relax condition. **(B)** Top: omnibus correlation between *RSP* values and time-of-night across all guided conditions. Error bar indicates 95% CI based on bootstrap resampling. Bottom: associated frequency pattern, represented with bootstrap ratios across conditions. Reliable positive (negative) bootstrap ratios are positively (negatively) correlated with time-of-night and are indicated by red (blue) circles. As the night progressed, there was as a gradual shift towards more power in high frequencies and less in low frequencies.

**Fig 9:**
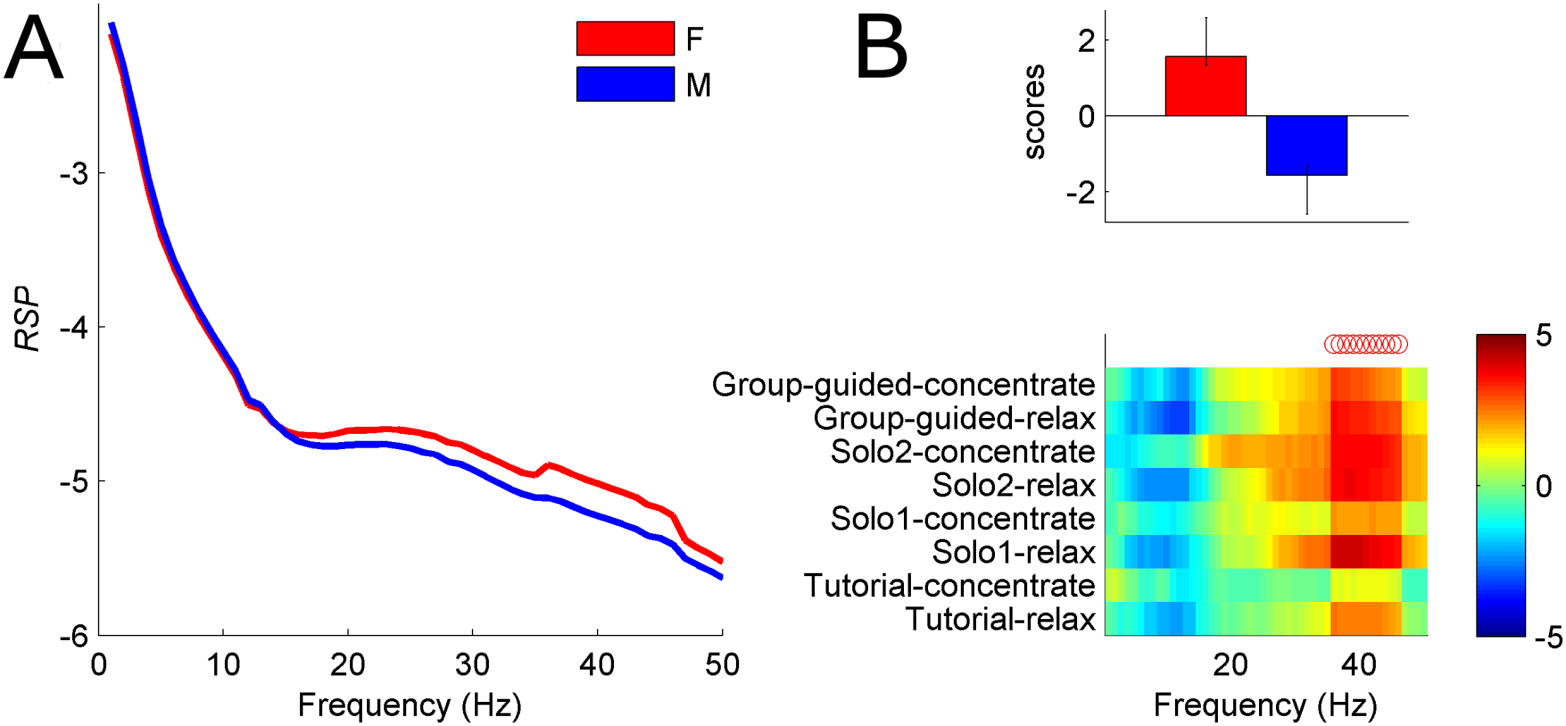
Sex differences in *RSP* (example left frontal channel). **(A)** Mean *RSP* curves for males and females during Solo2-concentrate condition. **(B)** Sex effect across all guided conditions. Top: mean participant scores with error bars representing 95% CI. Bottom: associated frequency pattern, represented with bootstrap ratios across conditions. Reliable positive bootstrap indicated by red (blue) circles identify frequencies (35–45 Hz) where females have more power compared to males. Weak trend by which males exhibit more power alpha range (blue bootstrap ratios) is not consistently reliable across conditions.

The time-of-night effect was statistically reliable and the results remained virtually unchanged after regressing out age. We found that as night progressed, the participants were showing a gradual shift towards more relative power in high frequencies and less in low frequencies (Fig 8). Initially we detected a relationship between *RSP* and age, however the effect disappeared after regressing the time-of-night effect. Notably, the correlation between time-of-night and age was -0.22 with 95 percent CI = [-0.13–0.31], meaning that older people participated mainly during earlier part of the night. We also detected sex differences whereby females showed more power in beta and gamma ranges (Fig 9).

## Discussion

By accessing a large number of participants we found subtle brain activity modifications that were taking place within approximately 1 minute of training, i.e., neurofeedback learning at a speed that has not been demonstrated before. Concentration training elicited a particularly salient effect of relative spectral power increase in beta range and decrease in frequencies < 8 Hz. The training effect was strong, yet subtle and could be detected only with large sample sizes (N >> 200). Besides overall power spectrum changes we also evaluated the actual neurofeedback performance. Approximately 3/5 of the participants showed steep beta neurofeedback learning across concentration blocks, with persistent changes affecting even relaxation blocks, i.e., periods without beta-based neurofeedback. The remaining participants showed opposite effect, such that their beta performance worsened with training. We further found that the two groups were significantly different at baseline, i.e., just before the onset of the neurofeedback training, in agreement with recent observations indicating that baseline states as measured by EEG predict learning [20], [21] and that regulation of those states via neurofeedback can modulate cognitive functions [33–35]. There is no explanation why the non-learners showed more power in beta/gamma range at baseline. It may imply that the non-learners were already very good at concentration at the beginning of the experiment. To better understand the reason for the difference between the two groups, the subjects’ ability of concentration at baseline could be tested in a future study.

Taking into account the novelty of the acquisition framework both in terms of the environment and the EEG-BCI technology, we performed a number of analyses in order to validate the data and reproduce effects that have been reported previously in the literature. Straightforward analysis of spectral power in response to neurofeedback instructions yielded the expected strong differentiation between ‘relax’ and ‘concentrate’ brain states (Fig 3) in terms of relative spectral power. Moreover, our results showed bias between frequency bands, which is common in neurofeedback, [36]. For example, concentration training targeted exclusively beta band, but was also accompanied by decrease in power for frequencies below 10Hz. In terms of global effects, we found a time-of-night effect, which can be interpreted as an extended wakefulness effect similar to findings in [37]. We also replicated previously reported sex differences [38], [39].

In conclusion, both confirmatory and novel findings from My Virtual Dream have provided necessary proof-of-concept for a novel neuroscience research framework. In our experimental design, we tested a tradeoff between the lack of formal control for confounding variables such as substance use and history of neurological problems, and access to massive sample size in a single session. Participants waited for 2 hours on average, although there were many other installations nearby with very little or no wait time. This fact, although not an explicit part of the design, can be taken as an indication that the participants were highly motivated. By combining art, performance and BCI we are now in a position to approach questions of complex real-life social cognition that otherwise are not accessible in lab settings. It has been pointed out that the traditional approach to studying the mind disregards the mind's central feature of being intrinsically subjective [40]. We believe that My Virtual Dream opens exciting new avenues for neuroscience research taking into account individuality, complexity and sociability of the human mind.

## Supporting Information

S1 Movie My Virtual Dream experience. Collage of photos and live footage.
(MP4)

S2 Movie Video capture of the game. (MP4)
